# The Health and Behavioural Effects of Individual versus Pair Housing of Calves at Different Ages on a UK Commercial Dairy Farm

**DOI:** 10.3390/ani11030612

**Published:** 2021-02-26

**Authors:** Sophie A. Mahendran, D. Claire Wathes, Richard E. Booth, Nicola Blackie

**Affiliations:** Royal Veterinary College, Pathobiology and Population Sciences, Hawkshead Lane, Hatfield, Hertfordshire AL9 7TA, UK; dcwathes@rvc.ac.uk (D.C.W.); rbooth@rvc.ac.uk (R.E.B.); nblackie@rvc.ac.uk (N.B.)

**Keywords:** calf, housing, individual, pair, growth rate, feed intake, novel object

## Abstract

**Simple Summary:**

The way in which dairy calves are housed can have a significant impact on their health and productivity. This study compared three different housing groups from birth to weaning; individual housing, pair housing from birth, and pair housing from three weeks of age. Newborn Holstein heifer calves (*n* = 100) were recruited over a six-month summer period from a single commercial dairy farm in the UK. Each calf had a weekly visit by the researcher over a 10-week period, where they were weighed and assessed for the presence of disease, along with measuring solid feed intake and the time to approach a novel object. Other management aspects including milk allocation were the same across groups. There was no effect of the housing group on average daily liveweight gain (ADLG), the presence of disease or the time taken to approach a novel object. The housing group did impact solid feed intake, with calves pair housed at either time period ingesting significantly more than individually housed calves. This study demonstrated that there were no detrimental effects on the health or growth of calves housed in pairs, with the added benefit of increased solid feed intake for pair housed calves, which is important for a smooth transition over the weaning period.

**Abstract:**

Housing management of dairy calves is one of the factors that contributes to a successful rearing outcome. Individual housing of pre-weaned calves is thought to provide enhanced biosecurity and easier monitoring of the individual, and so remains prevalent in the UK. Behavioural studies have, however, found that pair housing is important for social learning, with positive impacts on health and welfare. This study utilised a single UK commercial dairy farm to establish if individual housing, pair housing from birth, or pair housing from three weeks of age affected health and behavioural parameters. Calves were housed in these allocated groups from birth to eight weeks of age, when they were moved into group pens of five calves for weaning at 10 weeks of age. All management routines other than the housing group were the same for enrolled calves. One hundred Holstein calves were recruited over a six-month period, and systematically allocated to a housing group. Weekly visits were conducted up to 10 weeks of age (weaning) for each calf, with weight, solid feed intake, and presence of clinical disease measured. In addition, a novel object approach test was carried out at six weeks, and a thoracic ultrasound was performed at seven weeks. Housing group had no effect on the average daily liveweight gain (ADLG) (*p* = 0.74), with an average of 0.66 kg/day over the pre-weaning period. However, on group housing at 8–10 weeks of age, there was a numerical increase in ADLG in the pair housed calves compared to the individually housed calves over the weaning period. Housing group had no significant effect on disease prevalence (*p* = 0.98) or the time taken to approach the novel object (*p* = 0.29). However, pair housed calves had increased mean total solid feed intakes from weeks 2–8 (*p* = 0.011), with 6.2 ± 0.67 kg (standard error of the mean—SEM), 12.7 ± 0.73 kg and 13.6 ± 0.70 kg ingested by individually housed, pair housed from birth and pair housed from three weeks of age, respectively. The overall findings of this study indicate that within a UK commercial dairy management system, there is no detrimental effect of housing calves within pairs (either from birth or three weeks of age) compared to individual housing.

## 1. Introduction

Rearing dairy heifer calves capable of reaching their genetic potential for milk production requires excellent health along with good growth rates in order to achieve target ages for optimum first service and first calving. Heifers must then be sufficiently robust to thrive in the milking herd, but at present up to 19% of heifers in the UK leave the herd during their first lactation [1]. There are many management factors that contribute to successful rearing outcomes during the crucial pre-weaning period, with the main ones being colostrum protocols [2], nutritional regimens [3], and housing management [4].

Many calf housing recommendations over the previous two decades have focused on individual housing during the pre-weaning period, with reports of approximately 60% of UK herds using individual pens [5]. One of the main reasons for this was the perceived reduction in risk of transmission of enteric pathogens by faeco-oral transmission [6], and reduced risk of aerosol spread of respiratory pathogens [7]. In addition to lower disease prevalence, there were also perceptions of higher weight gains and reduced problems from cross-sucking for calves in individual pens [8]. However, these views have not been supported by more recent studies that have shown no difference in enteric or respiratory pathogen spread among pair housed calves [9].

Other studies have demonstrated the importance of social facilitation and social learning, whereby calves initiate specific behaviours (such as eating concentrates) while observing others engaged in that behaviour [10], and are thus influenced by observation or interaction with another individual [11]. This is borne out in behavioural studies, with pair housed calves spending more time at the feeder, visiting the feeder more often, and starting to ingest concentrate more rapidly than individually housed calves [12]. This then translates into improved weight gains in pair housed calves [12,13], which continue after the weaning period [14]. The presence of another calf also has a calming effect on behavioural responses in stressful situations [15,16,17], with individually reared calves shown to be more fearful when introduced to a novel social situation and when isolated in a novel arena [18,19]. On the other hand, pair housed calves have shown higher behavioural flexibility, being able to modify their behaviour in response to a changing environment such as mixing with unfamiliar calves [12]. This is thought to have long-term positive benefits that can translate into improved social skills as an adult cow in the milking herd [20].

The positive impact that pair housing has demonstrated on calf behaviour has shown varying dependency on the age at which the pairing began. Costa et al. [21] compared calves that were transferred from individual to pair housing at one week compared to six weeks of age, and found that the benefits of increased weight gain were only seen in the early-paired calves (0.89 vs. 0.73 kg/day). Duve and Jensen [22] examined the social behaviour of calves housed in pairs from birth compared to at three weeks of age, and found only minor differences in lying down times, with all other monitored behaviours (sniffing, licking, social contact, and play) demonstrating similar levels. This is also supported by Jensen et al. [9] who found that there was no difference in behavioural responses to a novel environment or exposure to a new calf between animals pair housed from birth compared to two weeks of age [9]. This suggests that social contact in the first few weeks of life are not essential for development of beneficial behavioural responses later in life [9].

Another area that impacts calf management is the public perception associated with different types of calf housing. In a survey comparing public opinions, over 75% of participants found group housing to be the most acceptable way to keep calves (compared to pair and individual housing), with their main reasons being that they thought it avoided isolation and provided more space [23]. The survey also found that the participants thought that pair housed calves had better opportunities for socialization and play interactions than individually housed calves [23].

Much of the body of evidence supporting the benefits of pair housing calves originates predominantly from research institutions (not commercial dairy units) that fed larger volumes of milk (between 10 L to ad-lib) [24] than are typically seen on many UK dairy farms [25]. They also often utilized bull calves, and calves were housed under different environmental (weather) conditions to those found in the UK. The aim of this study was to establish the effect of individual and pair housing at different ages on a commercial dairy farm, under environmental conditions and management practices more representative of the UK dairy sector.

## 2. Materials and Methods

### 2.1. Animals and Housing

This study was conducted on a single commercial dairy farm in the South-West of England, milking 550 Holstein dairy cows in an all year round calving pattern. A total of 100 heifer calves were recruited from March to August 2020, with all work conducted following social distancing guidelines. Calves were born in a loose housed straw yard, and were provided with two 3 L colostrum feeds from their own dam within 12 h of birth via an oesophageal feeding tube (quality was not measured). Calves were then transported to the rearing area and housed outdoors in commercially available calf hutches (Calf-Tel^®^, Hampel Corporation, Germantown, WI, USA) with internal hutch dimensions of 2.2 m long × 1.22 m wide × 1.38 m high.

**Figure 1:**
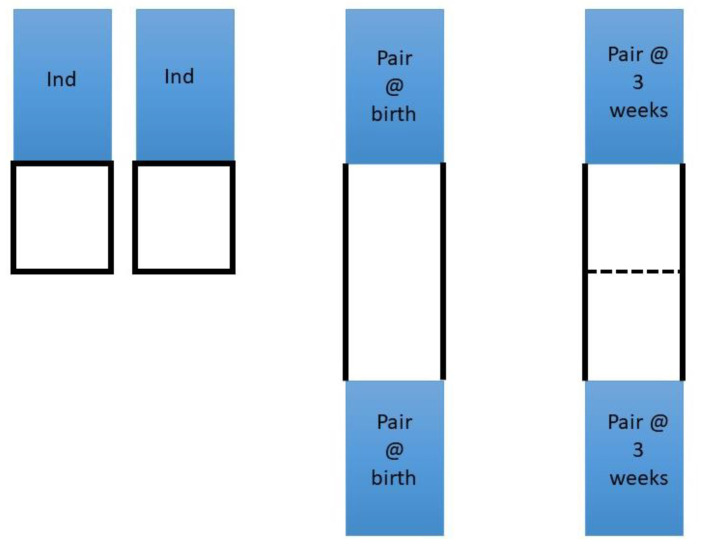
Diagram demonstrating the layout of the three different housing groups in the study. ‘Ind’ identifies the hutches for individual calves. The blue boxes indicate the calf hutch, the black solid lines indicate the metal partitions surrounding the outside area of the pen, and the black dashed line indicates the small internal gate used to initially separate calves that became pair housed at three weeks of age. The individual pens were placed next to each other so calves could only see each other when outside.

**Figure 2:**
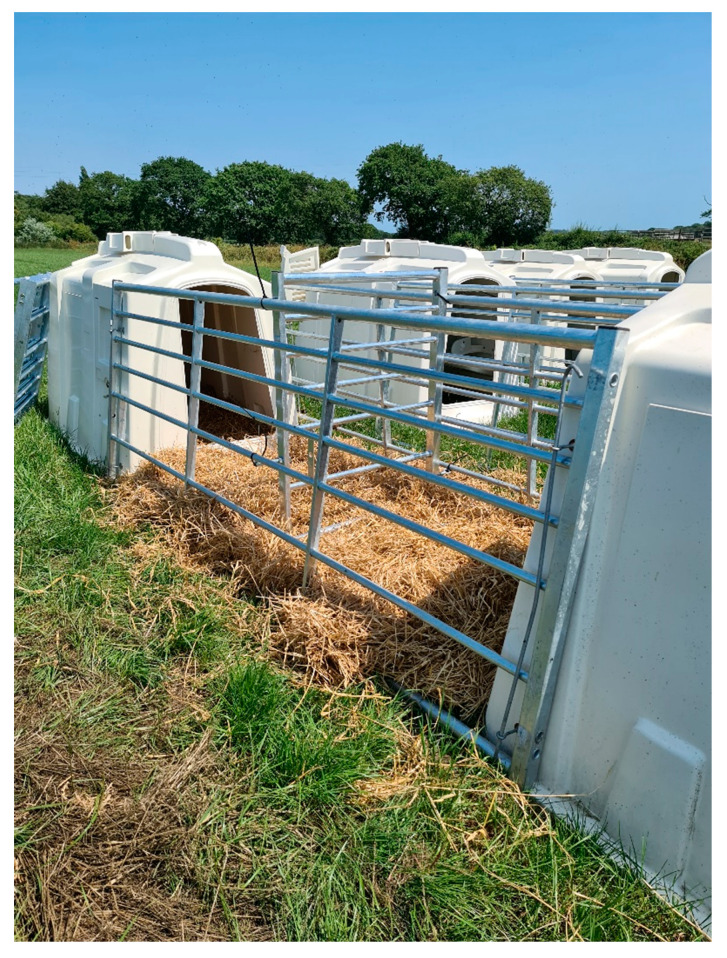
Image demonstrating the layout used for pair housing. For calves that were individually housed until three weeks of age (group c), a small partition gate was initially placed to separate the outdoor areas and create two pens. This was removed at three weeks of age. The calves housed in pairs from birth (group b) did not have the partition gate, therefore always had access to both hutches and the full outdoor area.

Three different pre-weaning housing systems were compared: (a) individual housing (*n* = 20), (b) pair housing from birth (*n* = 40) or (c) individual housing for the first three weeks, then subsequent pair housing (*n* = 40), with the layouts illustrated in Figure 1. (a) Individually housed calves were placed in a single hutch, with a wire mesh outdoor area measuring 1.5 m long × 1.22 m wide. These pens were arranged side by side, so that the calves could only see each other when in the outside area. They could, however, touch their neighbour if they placed their heads over the wire partitions. (b) Pair housed calves were provided with two hutches per pair, with the hutches facing each other and two 3 m gates between them used to make an outdoor area. (c) Calves paired at three weeks of age were initially housed in individual hutches, arranged as in (b) but with the outdoor area split by an internal gate. The pair were subsequently combined by removal of this internal gate (Figure 2). At eight weeks of age, all calves were moved from their allocated housing type into groups of five calves in group hutches, where they stayed until after weaning (at 10 weeks of age).

Calves born between March and May were systematically allocated at birth into housing groups (a) or (b). From April to August, all calves were allocated to group (c). The difference in recruitment time was due to expansion of the original study design, triggered by a changed management requirement imposed by some UK milk buyers which stipulated pair housing of calves at three weeks of age. All three groups were, however, born during the relatively warm spring/summer seasons. The effect of these different recruitment times was included in all the final models to check for potential confounding, as described below.

A sample size calculation was carried out during design of the study using published differences in growth rates between individually and pair housed calves of 0.13 g/day [21]. The variance was calculated as 0.10. Based on a confidence level of 0.95 and a power of 0.8, using a 2-tailed test, the sample size for detecting a significant difference between three treatment groups was *n* = 20/treatment [26].

### 2.2. Calf Nutrition

Each calf was fed a 22.5% whey protein and 25% oil, calf milk replacer (Advanced Optistart 25, Advanced Sourcing, Dunston, Staffordshire, UK) mixed at 13.5% concentration, fed through a teat feeder. The calves were fed a standardized regime starting at 3 L twice daily from day one to day fourteen, increasing to 3.5 L twice daily from day 14 to 21, and then increasing to 4 L twice daily from day 21 to 48. The calves were then step weaned down from day 49–70 by reducing the milk volume fed by 2 L per week. Each hutch had ad libitum water from a bucket, with forage provided by straw bedding which was refreshed daily.

Calves were provided with ad libitum pelleted concentrate, with 21% crude protein, 5.1% fats, 30.0% neutral detergent fibre (NDF), 33% starch and 12.5 MJ/kg (Rearer 21 nuts, Mole Valley, UK). This was provided within a bucket inside the hutch (one per calf). On two consecutive days each week, between weeks 2 to 8, the weight of the concentrates was measured to allow intakes to be calculated. In pair hutches, the weight was divided by two to provide an estimated intake per calf.

### 2.3. Performance and Health

Each calf underwent a weekly visit for a consecutive period of 10 weeks by the researcher (SAM). At each visit, the weight was measured using a weigh band (AHDB, Stoneleigh Park, Warks, UK) placed around the girth behind the forelimb. This method has previously been validated against actual weigh measurements [21]. Birthweight was taken as the measurement at the first visit between 0–7 days of age. Actual growth rates were calculated by subtracting the start from the end weight and dividing by the exact number of days between the two measurements. An average growth rate over the entire pre-weaning period was calculated, as well as over three time periods: 2 to 4 weeks, 5 to 7 weeks, and 8 to 10 weeks. This allowed for compatible comparisons between all calves, regardless of the exact age at each measurement [27].

Measurement of passive transfer was already routinely carried out as a management procedure on the study farm. Blood was sampled from the jugular vein into a plain vacutainer from calves between 2–8 days of age. The samples were left to stand for 24 h, before a sample of serum was placed onto a refractometer to assess serum total protein (TP).

At each visit, the calves underwent a clinical health assessment following a modified scoring system developed by the University of Wisconsin-Madison [28,29] which was modified to assess demeanour, nasal and ocular discharge, cough, faecal consistency, rectal temperature, navel and joint health on a scale of 0 to 3. This was then simplified to a binary classification of disease being either present (score 1) or absent (score 0) within the same three periods as for growth rates [30]. When calves were identified as ill during a visit, they were treated according to current veterinary practices adopted on farms by the farm staff.

Thoracic ultrasonography of all calves was carried out at seven weeks of age. After application of 70% isopropyl alcohol to each thoracic area of the calf, a 7.5 MHz linear transducer was used to assess both sides of the thoracic cavity for pathology [31]. A categorical scoring system was used to record lesions where Score 0 indicated normal aerated lung with none to few comet-tail (B- line) artefacts, Score 1 indicated diffuse comet tails but without consolidation and Score 2 indicated lobular or patchy pneumonia with consolidation [32].

### 2.4. Novel Object Appraoch

An open umbrella was used as a novel object, which was placed into each calf pen during the sixth visit. Prior to placement, it was ensured that the calves were standing up within the hutch, and the umbrella was placed into the outside area. The time was measured from placement of the umbrella until it was touched by the nose of a calf. In pair pens, the time was stopped when just one of the calves made contact with the umbrella. The calves were observed for a maximum time limit of 10 min, and if no contact was made, it was recorded as a non-approach [33].

### 2.5. Statistical Analysis

All data was stored in Excel (Microsoft Office; Microsoft, Redmond, WA, USA). All analyses were performed using SPSS (Version 27.0, IBM SPSS Statistics for Windows, NY: IBM Corp). Significance was declared at *p* ≤ 0.05, and trends were reported if *p* ≤ 0.10.

The outcomes of average daily liveweight gain (ADLG) over the three time periods and feed intake for ages 2 to 8 weeks old were analysed using linear mixed effects models. The overall fixed effects included were month of enrolment (to check for the effect of different recruitment times), housing group (individual, pair at birth and pair at three weeks), birthweight, total protein level, total mean concentrate intake, presence of disease, and ultrasound score. Pen and calf identification number were included as random effects. Results are reported as F-values in the format F_(treatment df, error df)_. For all analyses, the assumption of normality was assessed through visual inspection of residual plots.

The outcome of disease occurrence was analysed using binary logistic generalised estimating equations, with the variable pen used to account for repeated measures within a pair of calves. The dependent variables were month of enrolment, housing group (individual, pair at birth and pair at three weeks), birthweight, total protein level, and total feed intake.

The outcome of novel object approach time was analysed by generalised linear model, with pen used as the experimental unit, and the variables of month of enrolment, housing group and interaction between month of enrolment and housing group. A Chi square analysis was carried out to compare the number of none approaches to the novel object between different housing groups.

## 3. Results

One hundred Holstein heifer calves were recruited into the study over a six-month period. During the study, two calves died (one individually housed, and one in the pair at three week group), giving a 2% mortality rate. Cause of death was unknown. Data from both calves and the associated pair were excluded from analysis, leaving 97 calves in the study analysis.

### 3.1. Weight Gain

**Figure 3:**
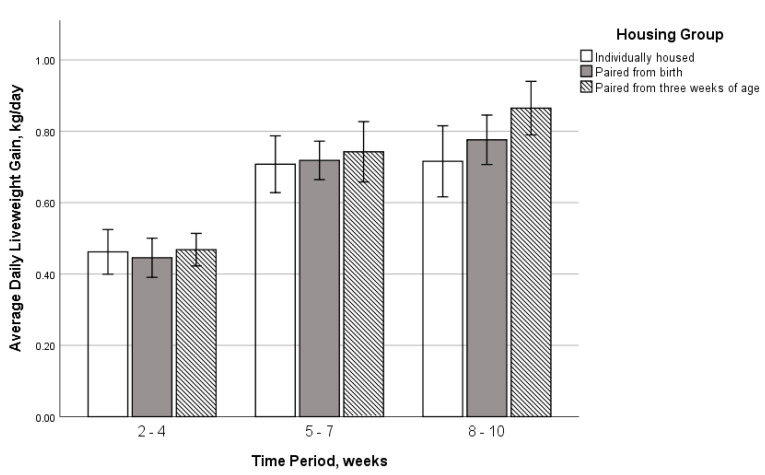
ADLG (kg/day) over the different time periods for the three different housing groups, with the 95% confidence interval. Calf numbers for each group include individually housed calves (*n* = 19 calves), calves paired at birth (*n* = 40 calves), and calves paired at three weeks of age (*n* = 38 calves). All calves were housed in groups of five during weaning (weeks 8–10).

The ADLG of the calves within the three time periods was not affected by the housing group (F_2,274_ = 0.30, *p* = 0.74; Figure 3), with a mean ADLG in weeks 2–4 of 0.46 ± 0.02 kg/day, in weeks 5–7 of 0.73 ± 0.02 kg/day, and in weeks 8–10 of 0.80 ± 0.02 kg/day. The overall ADLG across weeks 1–10 was 0.66 ± 0.01 kg/day (standard error of the mean—SEM) (range 0.35–0.97 kg/day). There was a non-significant numerical difference over the weaning period (weeks 8 to 10), with housing group (a) achieving 0.72 ± 0.05 kg/day (SEM), group (b) achieving 0.78 ± 0.03 kg/day, and group (c) achieving 0.86 ± 0.04 kg/day. This suggested a tendency for pair housed calves to have a greater average increase in weight gain compared to individually housed calves, even though all calves were group housed over this period.

The average birthweight of the calves was 42 ± 0.18 kg (SEM) (range 36–48 kg), and this had a significant effect on the ADLG (F_1,274_ = 5.00; *p* = 0.026), with a 1 kg increase in birthweight resulting in an 0.011 kg increase in ADLG. There was no significant effect of the month of enrolment (F_5,274_ = 1.05; *p* = 0.39), indicating that the different periods of enrolment for the housing groups had no effect on treatment outcome. There also was no significant effect of passive transfer (measured as serum TP) (F_1,274_ = 1.59; *p* = 0.21), total concentrate feed intake between weeks 2 to 8 (F_1,274_ = 0.075; *p* = 0.79), the occurrence of disease (F_1,274_ = 2.46; *p* = 0.12), or of the thoracic ultrasound score for the calf (F_2,274_ = 0.84; *p* = 0.43).

### 3.2. Concentrate Feed Intake

**Figure 4:**
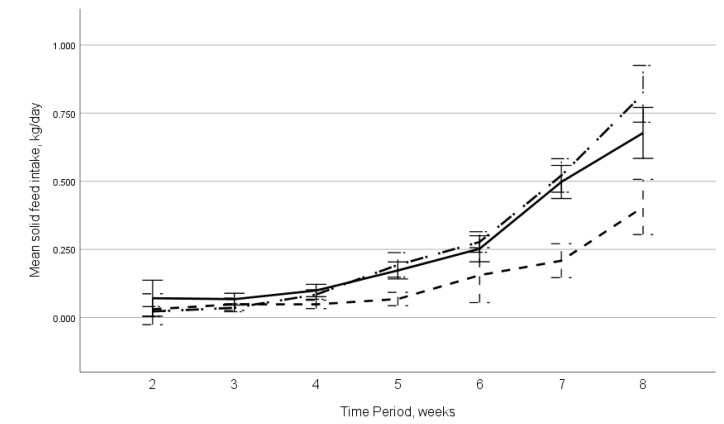
Mean measured concentrate feed consumption between visits at 2 to 8 weeks of age for the different housing groups of calves, with the 95% confidence interval shown as error bars. The 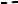 line indicates the individually housed calves (*n* = 19 calves), the 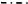 line indicates the calves paired at birth (*n* = 40 calves), and 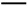 line indicates the calves paired at three weeks of age (*n* = 38 calves).

The housing group had a significant effect on the amount of concentrate feed ingested by the calves over weeks 2 to 8 (F_2,566_ = 4.56; *p* = 0.011, Figure 4), with an estimated mean total of 6.2 ± 0.67 kg (SEM), 12.7 ± 0.73 kg, and 13.6 ± 0.70 kg ingested by individually housed, pair housed from birth, and pair housed from three weeks of age, respectively. Most of this difference occurred between weeks 5–8 as consumption increased over time.

There was no significant effect of month of enrolment (F_5,566_ = 1.00; *p* = 0.42), birthweight (F_1,566_ = 1.14; *p* = 0.29), or occurrence of disease (F_1,566_ = 1.05; *p* = 0.31), which were all included in the final model. 

### 3.3. Passive Transfer

The week 1 total protein levels in blood ranged from 3.8–8.2 g/dL, with 85% of calves classed as having good passive transfer as indicated by a level of ≥5.2 g/dL [34]. There was no difference between the three housing groups.

### 3.4. Disease Occurrence

A total of 38 calves (39.1%) experienced disease during the pre-weaning period (Table 1), with cough and diarrhoea being the most common presenting clinical signs. There was no significant effect of housing group (*p* = 0.98), month of enrolment (*p* = 0.18), blood total protein level (odds ratio (OR) = 1.01 (0.94–1.08); *p* = 0.78), birthweight (OR = 0.97 (0.82–1.15); *p* = 0.72), ADLG (OR = 0.013 (0.004–4.53); *p* = 0.15), or total concentrate feed intake (OR = 0.63 (0.33–1.18); *p* = 0.15) on the occurrence of disease. The data did, however, demonstrate a tendency for an association between ultrasound score and the concentrate feed intake (F_2,566_ = 2.47; *p* = 0.085), with an estimated total concentrate intake of 12.8 ± 0.58 kg (SEM), 9.9 ± 1.00 kg and 6.6 ± 1.36 kg for thoracic ultrasound scores of 0, 1, and 2 respectively. This suggested a potential association between lung disease and reduced feed intakes. 

### 3.5. Novel Object Approach

**Figure 5:**
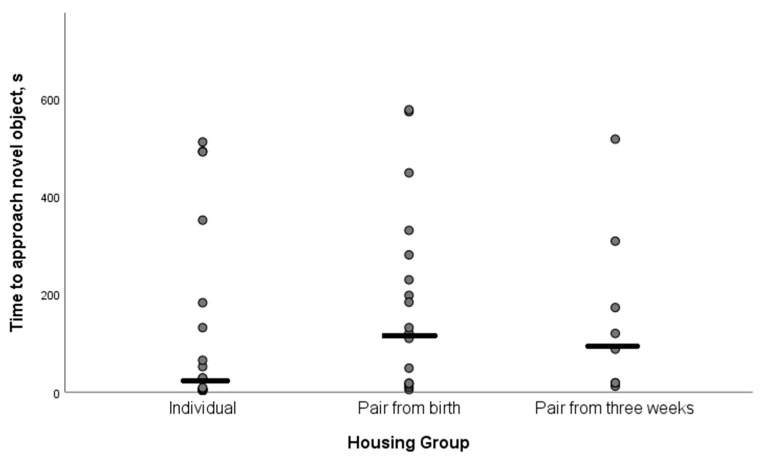
A scatter graph demonstrating the spread of time taken to approach the novel object for each housing group of calves. Each dot represents either an individual calf in the individually housed calf group, or the time taken for the first calf to touch the novel object in the pair housed groups. The horizontal line shows the median time for that group to approach the object.

This test was performed during the visit of week 6. Of all the calves observed, 1/19 (5.3%) individual, 3/20 (15.0%) paired at birth and 11/19 (57.9%) paired at three weeks of age did not approach the novel object within the observation time limit of 10 min. A χ^2^ analysis demonstrated a significant difference in no approaches between the groups (*p* < 0.01). Of the calves that did approach, there was no significant effect of housing group (*p* = 0.29) or any interaction between housing group and month of enrolment (*p* = 0.31) on the time taken to approach and touch the umbrella (Figure 5). The mean time to approach the novel object was 177 ± 23.3 s (SEM). The month of enrolment demonstrated a tendency towards being associated with the time to approach the novel object (*p* = 0.066), which was between 10–35 s less in the calves paired at 3 weeks than groups (a) and (b). This result needs, however, to be interpreted with caution as only eight calves in this group did approach the novel object.

## 4. Discussion

This study examined the effects of individual and pair housing of calves at different ages on a commercial dairy farm to establish if existing research findings were applicable under commercial management and environmental conditions in the UK. 

### 4.1. Weight Gain

During the pre-weaning period from 1 to 10 weeks of age, the ADLG of the calves was not affected by the housing group (*p* = 0.74), with the ADLG being 0.66 ± 0.098 kg/day (SD) over the entire period. This is in agreement with other reported figures [35]. The lack of association with housing group size is also in agreement with other studies [8,33,34,35,36], indicating no negative impact on pair housing of calves. However, the ADLG was below the minimum requirements of 0.7 kg/day growth needed for an age at first calving target of 24 months [37,38]. The relatively low ADLG may be due to the restricted milk feeding protocols used, which are relatively common across UK dairy farms [39]. This low milk feeding level affects concentrations of insulin-like growth factor 1 (IGF-1), which helps with growth promotion; therefore, low levels from restricted feeding rates are linked to reduced growth rates in calves [40].

Although the overall ADLG did not differ between housing groups, there was a numerical tendency towards higher growth rates of around 60 g/day in the calves which were pair housed at three weeks of age. Although this finding may have been due to chance, the study was underpowered to find a difference of less than 130 g/day in growth rates between housing groups [21]. For the difference seen to become significant, a sample size of 90 calves per housing group would have been required. To achieve this on a single farm, a recruitment period much longer than the six months used in this study would have been needed, increasing the likelihood of seasonal effects due to temperature differences. 

The ADLG was also assessed separately over three time periods, with poor growth seen in weeks 2 to 4 of life, reaching only 0.46 ± 0.02 kg/day (SEM), and improving in the second month of life, reaching a mean 0.80 ± 0.02 kg/day. These relatively low growth rates early in life have been found in other studies [27,39,41], and represents a large loss in potential growth efficiency due to the excellent feed conversion that young calves are able to achieve. There was a numerical difference in ADLG over the weaning period (weeks 8 to 10), with the calves pair housed from three weeks of age having a greater average increase in weight gain (0.86 ± 0.23 kg/day (SEM)) compared to individually housed calves (0.72 ± 0.21 kg/day (SEM)), even though all calves were group housed over this period. Other studies such as Chua et al. [8] have reported significant reductions in growth rate in response to weaning, potentially caused by the transitioning into group pens being stressful due to both the physical handling and movement, the introduction to a new environment, and meeting new calves. Calves that are initially pair housed have been shown to cope better with stress through the benefits of social support [42], with individually housed calves being more reactive to unfamiliar calves [43], which can have a negative impact on feed intakes and therefore growth rates. Calves pair housed at three weeks of age may benefit from a lack of competition for milk resources in the first few weeks of life, combined with the ability to interact with a peer once slightly stronger, thus still benefitting from social learning. Knauer et al. [36] found that pair housed calves had greater weight gain pre-weaning, and this continued with a small numerical increase in bodyweight up to 16 weeks of age. This indicates that the early benefits associated with pair housing may continue longer term, and future studies on early life housing should continue to monitor calves post-weaning to establish if this difference persists in calves that are pair housed at three weeks of age.

Calf birthweights ranged from 36–48 kg, with a mean value of 41.7 kg and no difference between the housing groups. However, the average birthweight of the calves did have a significant effect on the ADLG that the calf was able to achieve (*p* = 0.026), with a 1 kg increase in birthweight resulting in a 0.011 kg increase in ADLG. This may be due to larger calves being stronger and more competitive for feed, which is in agreement with some literature that larger calves have greater pre-pubertal growth rates compared to smaller calves [39,44]. However, it is in contrast to another study, which found that calves with smaller heart-girth circumferences had compensatory increases in ADLG [45]. This may only be possible when higher milk feeding rates are used. There may also be an effect of dam parity, with smaller calves born to primiparous dams able to exhibit catch-up growth [38], whereas small calves born to multiparous dams were not [44].

There was no effect of the month of birth on ADLG (*p* = 0.39), indicating that the difference in enrolment periods for the housing groups did not have an effect on the overall outcomes measured. In addition, the whole study was conducted over the spring/summer period in the UK, when average temperatures are in the range 12–26 °C. There was no effect of disease occurrence on ADLG (*p* = 0.12), which is in contrast to other studies that typically found that calves with disease had reduced growth [46,47,48,49]. The level of disease on this study farm was relatively low, with recorded clinical signs generally being mild, which may have limited the impact that disease occurrence had on ADLG. This may also be related to the season, as warmer weather in the UK was previously associated with a reduced incidence of BRD [50].

### 4.2. Concentrate Feed Intake

Calves between two and eight weeks old that were housed in pairs had increased concentrate feed intake (*p* = 0.011), consuming almost twice as much concentrate (12.7 ± 0.73 kg (SEM) for those paired at birth and 13.6 ± 0.70 kg for those paired at three weeks of age) compared to individually housed calves (6.2 ± 0.67 kg). This is similar to other studies that found increased solid feed intakes in socially housed calves due to social facilitation, with a calf more likely to approach a feeder when another calf is feeding [19,36,51,52,53], spending longer time periods eating [54], and in more frequent meals [55], with these differences known to continue in the post-weaning period.

Despite the difference in feed intakes between housing groups in this study, there were no significant differences in growth rates in the pre-weaning period (*p* = 0.74), although as mentioned above, there was a numerical trend towards higher growth rates in the pair housed calves around the time of weaning, which could be explained by the increased solid feed intakes. This lack of a significant difference in growth rates despite the significant difference in feed intake may be due to an insufficient sample size, as discussed above. Alternatively it may suggest differences in feed efficiency between the housing groups. One potential reason for this is that pair housed calves have been shown to be more active, so the extra feed intakes may have been used for activity rather than growth [53]. However, this was not recorded in this study.

In all housing groups, there were very low concentrate intakes up to four weeks of age, but this continued in the individually housed calves up until the point that weaning began (eight weeks of age). This may negatively impact on future feed intakes in individually housed calves, as feeding patterns acquired early in life can persist, potentially impacting production parameters for reproduction and lactation [55]. It should be noted that the individual housing style for calves that were paired at three weeks of age was different to those calves who were individually housed throughout the study (Figure 1). The layout of the hutches enabled paired calves to see directly inside the other hutch even when initially separated by a gate. This might have had an influence on development of behaviours in the first three weeks of life, but this was not assessed in this study.

The study indicated a tendency for an association between thoracic ultrasound scores and the concentrate feed intakes (*p* = 0.085), with estimated total concentrate intakes of 12.8 ± 0.58 kg (SEM), 9.9 ± 1.0 kg, and 6.6 ± 1.36 kg during weeks two to eight, for thoracic ultrasound scores of 0, 1, and 2, respectively. This did not, however, result in a significant effect on ADLG, although Cramer et al. [56] demonstrated a reduction in growth of calves with lung consolidation. We were unable to determine whether calves that consumed less food (for any reason) were more likely to experience respiratory disease or vice versa. In addition, lung consolidation is not always associated with clinically observable changes [57], which may explain the relatively low level of clinical respiratory disease identified in the calves during the scoring process. 

### 4.3. Disease Occurrence

A common perception by farmers is that social housing of calves results in higher disease rates, but this study indicated no significant effect of pair housing (*p* = 0.98) on disease occurrence. This is in agreement with other studies [8,9,42], and confirms there is no detrimental effect of pair housing on calf health. This may be because contact between individually housed calves was still possible (in compliance with the EU directive 97/2/EC), allowing both faecal-oral and aerosol transmission of pathogens between pens, producing little difference to the pair housed calves. There are reports of increased disease prevalence in larger group sizes, although this is likely to be due to mixing of calves of different ages and sharing of single teats when automatic calf feeders are used [58,59,60].

The disease prevalence in the study was 39.1%, with the most prevalent clinical signs being a cough (17.5%) and diarrhoea (12.4%). This disease prevalence is lower than in other UK studies [27,61]. The presence of a cough without other clinical signs may have been indicative of Bovine Respiratory Disease [BRD]. The use of weekly calf health scoring meant that we were potentially able to identify affected calves either early in the disease course or with only mild signs [56,62]. However, on this farm, a higher sensitivity to clinical signs did not translate into an increase in calf treatments. The high relative proportion of calves with loose faeces is supported by other studies, suggesting this is a common occurrence in young calves [8,9].

### 4.4. Novel Object Approach

There were no significant effects of the housing group (*p* = 0.29) on the time taken to approach the novel object. However, the number of calves that did not approach the novel object within 10 min of it being placed in the pen was significantly different between the housing groups (*p* < 0.01). Only one individually housed calf (5%) failed to approach compared with 11 (58%) of the calves paired at three weeks of age. Researcher observations indicated that a large proportion of the calves paired at three weeks entered a lying down position and appeared to ignore the novel object. This may suggest that individually housed calves were more willing to explore their environment, which is supported by the theory that individually reared animals show enhanced effects of reward-related stimuli [63]. These findings are in contrast to some other studies, which have shown that individually housed calves are more fearful and reluctant to approach novel objects [9,18], and back off during exploration, which may be an indicator of heightened anxiety [43]. The individually housed calves in this study were, however, able to have tactile contact with each other through the outdoor pen fencing, which has been shown to reduce fearfulness [12].

## 5. Conclusions

This study aimed to assess different calf housing strategies within a commercial dairy management system, under typical UK environmental conditions. Overall findings indicate that there were no detrimental effects of housing calves within pairs (either from birth or from three weeks of age) compared to individual housing. This was shown by no significant differences in average daily liveweight gain, disease prevalence, or novel object approach times between the housing groups. However, we did find increased solid feed intakes in pair housed calves, which may have long-term benefits on calf development.

## Figures and Tables

**Table 1:** Distribution of the disease occurrence and thoracic ultrasound score during the pre-weaning period between the different housing groups of calves. For a calf to be diagnosed as having Bovine Respiratory Disease (BRD), they must have had a raised rectal temperature (≥39.5 °C) and either a cough or ocular or nasal discharge. An ultrasound score of 2 indicated lobular or patchy pneumonia with consolidation.

Clinical Sign	Individual	Pair Housedfrom Birth	Pair Housed at 3 Weeks	Total
Bovine Respiratory Disease	0	0	4	4 (4.1%)
Cough	5	5	7	17 (17.5%)
Diarrhoea	1	7	4	12 (12.4%)
Diarrhoea and Cough	1	0	1	2 (2.1%)
Diarrhoea and Nasal Discharge	0	0	1	1 (1.0%)
Diphtheria	0	1	0	1 (1.0%)
Nasal Discharge	1	0	0	1 (1.0%)
No Disease	11	27	21	59 (60.9%)
Thoracic ultrasound score 2	2	0	3	5 (5.2%)
